# Impact of Chronic Renal Failure on Safety and Effectiveness of Paclitaxel-Eluting Stents for Femoropopliteal Artery Disease: Subgroup Analysis from Zilver PTX Post-Market Surveillance Study in Japan

**DOI:** 10.1007/s00270-017-1673-6

**Published:** 2017-05-09

**Authors:** Yukihisa Ogawa, Hiroyoshi Yokoi, Takao Ohki, Kimihiko Kichikawa, Masato Nakamura, Kimihiro Komori, Shinsuke Nanto, Erin E. O’Leary, Aaron E. Lottes, Alan T. Saunders, Michael D. Dake

**Affiliations:** 1 Department of Radiology, St. Marianna University School of Medicine, Kawasaki, Japan; 2 Department of Cardiovascular Medicine, Fukuoka Sanno Hospital, Fukuoka, Japan; 3 Department of Surgery, Jikei University School of Medicine, Tokyo, Japan; 4 Department of Radiology, Nara Medical University, Kashihara, Japan; 5 Division of Cardiovascular Medicine, Ohashi Medical Center, Toho University, Tokyo, Japan; 6 Division of Vascular Surgery, Department of Surgery, Nagoya University Graduate School of Medicine, Nagoya, Japan; 7 Department of Cardiology, Nishinomiya Municipal Central Hospital, Nishinomiya, Japan; 8 Cook Research Incorporated, West Lafayette, IN USA; 9 Department of Cardiothoracic Surgery, Falk Cardiovascular Research Center, Stanford University School of Medicine, 300 Pasteur Drive, Stanford, CA 94305-5407 USA

**Keywords:** Drug-eluting stent, Paclitaxel-eluting stent, Peripheral artery disease, Femoropopliteal artery, Chronic renal failure

## Abstract

**Purpose:**

Favorable long-term outcomes of the Zilver PTX drug-eluting stent (DES) in femoropopliteal lesions have been demonstrated. Chronic renal failure (CRF) has been shown to be a risk factor for restenosis and decreased limb salvage. The results of the DES in patients with CRF have not previously been reported. This study compares the results with the DES in patients with CRF and those without CRF.

**Methods:**

This retrospective analysis from the Zilver PTX Japan Post-Market Surveillance Study included 321 patients with CRF and 584 patients without CRF. Outcomes included freedom from target lesion revascularization (TLR) and patency.

**Results:**

Of the patients included in this subgroup analysis, 2-year data were available for 209 patients in the CRF group and 453 patients in the non-CRF group. The two groups were similar in terms of lesion length and the frequency of in-stent restenosis. Critical limb ischemia, severe calcification, and diabetes were more common in patients with CRF, whereas total occlusion was more common in patients without CRF. Freedom from TLR rates were 81.4 versus 84.9% (*p* = 0.24), and patency rates were 70.7 versus 70.3% (*p* = 0.95) in patients with and without CRF at 2 years, respectively.

**Conclusion:**

This is the first comparative study of the DES in femoropopliteal artery lesions in patients with and without CRF. These results indicate that the DES placed in femoropopliteal artery lesions of CRF patients is safe and effective with similar patency and TLR rates to patients without CRF.

**Level of Evidence:**

Level 3, Post-Market Surveillance Study.

## Introduction

Peripheral arterial disease (PAD) is commonly seen in patients with chronic renal failure (CRF) including dialysis [1, 2]. Endovascular therapy is currently considered a first line of therapy for most cases of PAD when anatomically feasible [3]. There are many reports of bare metal stent (BMS) placement for treatment of patients with PAD involving the femoropopliteal (FP) arteries [3–6]. Throughout these experiences, CRF has been shown to be a significant risk factor for restenosis and decreased limb salvage [4, 5].

More recently, large clinical trials in patients with PAD have reported that a drug-eluting stent (DES) is able to reduce restenosis and provides superior long-term outcomes relative to BMS placement [7–11]. However, the effectiveness of the DES in PAD patients with CRF has not been established. The Zilver PTX Japan Post-Market Surveillance Study enrolled a large number of patients with CRF [12]. Taking advantage of this real-world population, a subgroup analysis compared the safety and effectiveness of the DES in patients with CRF to those without CRF.

## Methods

### Study Design

The current study is a subgroup analysis from the multicenter, prospective, single-arm Zilver PTX Post-Market Surveillance Study in Japan, with follow-up ongoing through 5 years [12]. The Zilver PTX DES (Cook Medical, Bloomington, IN) is a self-expanding nitinol stent with a polymer-free paclitaxel coating (3 μg/mm^2^ dose density). This study was required and regulated by the Japanese Ministry of Health, Labour, and Welfare, and informed consent processes were determined by each institution’s ethical committee policy.

A detailed description of the DES, study design, indication for patient treatment, and statistical analysis has been previously reported [12].

### Patient Population

A total of 905 patients with 1080 FP lesions were enrolled in this study between May 2012 and February 2013. Patients were divided into those with CRF, defined as an estimated glomerular filtration rate (eGFR) <60 mL/min/1.73 m^2^ and/or dialysis (*n* = 321, CRF group), and those without CRF (*n* = 584, non-CRF group).

### Baseline Assessment, Intervention, and Medication

Rutherford classification and ankle brachial index (ABI) were assessed pre-procedure. The device instructions for use recommend that the stent should be oversized by 1–2 mm with respect to the reference vessel and placed at least 1 cm below the superficial femoral artery origin and above the medial femoral epicondyle. Treatment of both legs was permitted. Pre- or post-dilatation and treatment of inflow or outflow disease were at the physician’s discretion.

The same antiplatelet regimen described in previous studies was recommended for all patients [7–10]. In general, this included clopidogrel or ticlopidine starting at least 24 h before the procedure, or a procedural loading dose, continued clopidogrel or ticlopidine therapy for at least 60 days post-procedure, and aspirin indefinitely.

### Follow-Up Assessment

Rutherford classification and ankle brachial index (ABI) were assessed at 1 year post-procedure.

Target lesion revascularization (TLR) was defined as re-intervention performed for ≥50% diameter stenosis within ±5 mm of the target lesion accompanied by recurrent clinical symptoms of PAD. Patency was assessed by duplex ultrasonography at 1 and 2 years where physicians considered this standard of care, with loss of patency corresponding to a peak systolic velocity ratio ≥2.4.

Stent thrombosis was site-reported as total occlusion of suspected thrombotic origin. Stent integrity was evaluated by radiography at 1 year, with the next evaluations planned at 3 and 5 years. Clinical benefit was defined as freedom from persistent or worsening symptoms of ischemia (i.e., claudication, rest pain, ulcer, or tissue loss) after the initial study treatment. Amputation rate was also assessed during follow-up periods.

### Statistical Analysis

The sample size of 900 was selected to provide 95% confidence for determination of events at rates as low as 1–2%. Continuous variables were summarized with means ± standard deviations, with *p* values calculated using the standard *t* test. Dichotomous and polytomous variables were reported as counts and percentages, with *p* values calculated using the Fisher exact test. Rutherford *p* values were calculated using the Cochran–Armitage test for trend. As appropriate, the number of observations represented the number of patients, treated lesions, and treated limbs. Kaplan–Meier analyses were used to assess freedom from TLR, freedom from thrombosis, clinical benefit, and patency over time, and log-rank test was used to compare the survival curves of the CRF and non-CRF groups. All data were analyzed using SAS software (version 9.3; SAS Institute, Inc, Cary, NC, USA).

## Results

**Fig. 1:**
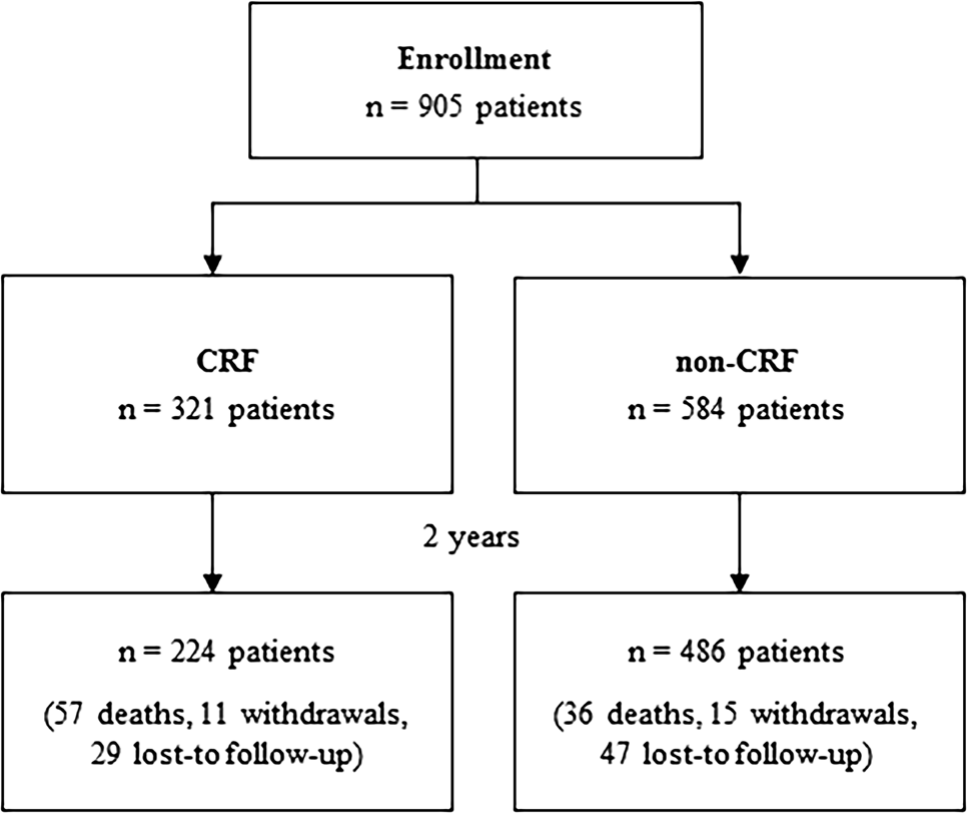
Patient flowchart. Of the 905 patients enrolled in the study, 321 patients were in the CRF group and 584 patients were in the non-CRF group. At 2 years, 224 patients in the CRF group and 486 patients in the non-CRF group remained in the study; death (all-cause), withdrawals, and lost to follow-up through 2 years are shown

Of the 905 patients treated with the DES, 321 (35.5%) patients were in the CRF group and 584 (64.5%) patients were in the non-CRF group. Demographics and lesion characteristics are shown in Table 1. Diabetes, critical limb ischemia, calcification, and reduced runoff were more frequently seen in the CRF group. In contrast, total occlusions were more prevalent in the non-CRF group. Other comorbidities and lesion characteristics including pre-procedure ABI, mean lesion length (145.8 ± 93.1 and 147.3 ± 98.2 mm), and the existence of in-stent restenosis were not significantly different between CRF group and non-CRF group, respectively. Of the 224 patients in the CRF group and 486 patients in the non-CRF group eligible for 2-year follow-up, data were available for 209 (93.3%) and 453 (93.2%) patients, respectively (Fig. 1).

**Table 1:** Patient demographics and lesion characteristics

	CRF	Non-CRF	*p* value
Patient, *N*	321	584	–
Age	72.1 ± 8.8 (321)	74.2 ± 8.2 (584)	<0.001
Male	67.9 (218)	71.6 (418)	0.25
Diabetes	69.2 (222)	53.1 (310)	<0.001
Hypertension	85.7 (275)	85.3 (498)	0.92
Hypercholesterolemia	56.7 (182)	63.0 (368)	0.06
Pulmonary disease	5.9 (19)	9.2 (54)	0.10
Lesions, *N*	381	699	–
Lesion length (mm)	145.8 ± 93.1 (381)	147.3 ± 98.2 (698)	0.8
Total occlusion	34.4 (131)	45.4 (317)	<0.001
In-stent restenosis	16.8 (64)	19.6 (137)	0.26
% diameter stenosis	91.3 ± 10.4 (381)	92 ± 11.1 (699)	0.29
Calcification			
None	14.2 (54)	33.6 (235)	<0.001
Mild	28.9 (110)	37.6 (263)	
Moderate	24.7 (94)	19.6 (137)	
Severe	32.3 (123)	9.2 (64)	
Rutherford^a^			
0	1.1 (4)	0.9 (6)	<0.001
1	6.3 (23)	7.8 (51)	
2	19.3 (70)	30.6 (201)	
3	39.9 (145)	46.0 (302)	
4	13.5 (49)	8.5 (56)	
5	17.4 (63)	5.5 (36)	
6	2.5 (9)	0.8 (5)	
Runoff vessels^b^			
0	6.9 (26)	6.5 (45)	0.19
1	35.3 (133)	30.1 (210)	
≥2	57.8 (218)	63.4 (442)	
ABI	0.63 ± 0.21 (339)	0.63 ± 0.16 (641)	0.69

Values are mean ± SD or % (*n*)

*ABI* ankle brachial index

^a^Rutherford classification data not available for 18 lesions in the CRF group and for 60 lesions in the non-CRF group

^b^Runoff vessel data not available for four lesions in the CRF group and two lesions in the non-CRF group

### Safety

**Fig. 2:**
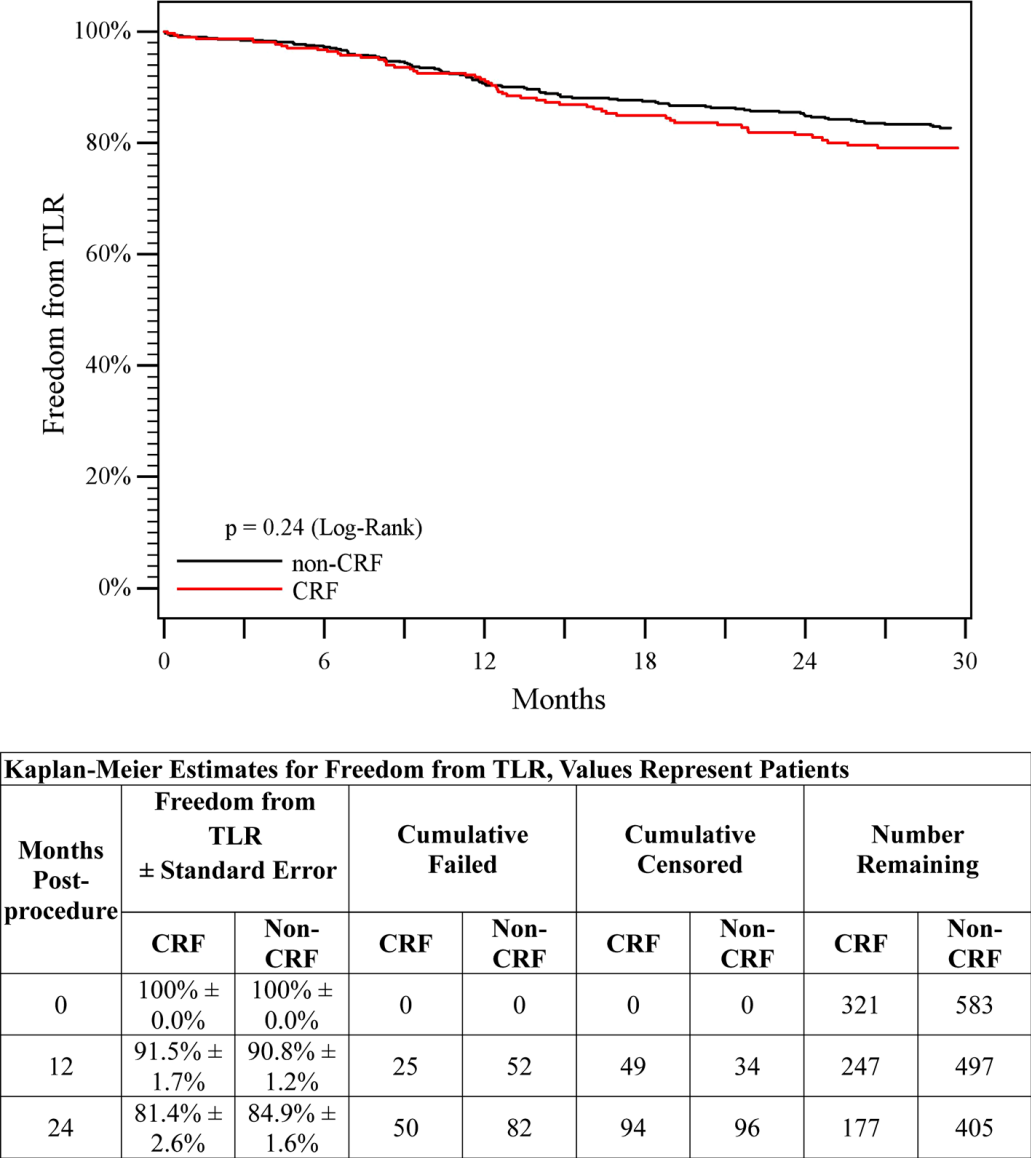
Freedom from TLR. Kaplan–Meier curves of freedom from clinically driven target lesion revascularization (TLR) for patients in the non-CRF group (*black line*) versus patients in the CRF group (*red line*). Freedom from TLR was 84.9% in the non-CRF group versus 81.4% in the CRF group through 2 years (*p* = 0.24, log-rank test)

There were no device- or procedure-related deaths. All-cause mortality through 2 years was 18% in the CRF group and 6% in the non-CRF group (*p* < 0.01). No paclitaxel-related adverse events were observed. A total of 1249 DES were implanted (438 in the CRF group and 811 in the non-CRF group). There were no perioperative stent fractures. At 1 year, one stent fracture (0.5%) was observed in the CRF group and 16 fractures (3.7%) in the non-CRF group (*p* = 0.016). As shown in Table 2, there were no significant differences in freedom from TLR or thrombosis through 2 years. A Kaplan–Meier curve for freedom from TLR is provided in Fig. 2, with 2-year rates of 81.4% for the CRF group and 84.9% for the non-CRF group (*p* = 0.24 log-rank). Through 2 years, eight patients (2.5%) in the CRF group and two patients (0.3%) in the non-CRF group had an amputation (*p* = 0.005). Of these, six patients in the CRF group and both patients in the non-CRF group had a pre-procedure Rutherford classification of five. Additionally, three amputations in the CRF group and one amputation in the non-CRF group occurred within 2 months from the intervention.

**Table 2:** Study outcomes

Outcome	Time	CRF	Non-CRF	*p* value
Stent fracture	1-year	0.5% (1/216)	3.7% (16/434)	0.016
Freedom from TLR	1-year	91.5%	90.8%	0.24^a^
	2-years	81.4%	84.9%	
Freedom from thrombosis	1-year	96.9%	96.6%	0.46^a^
	2-years	95.1%	96.3%	
Amputation^b^	1-year	2.2% (7/321)	0.3% (2/584)	0.01
	2-years	2.5% (8/321)	0.3% (2/584)	0.005
Patency	1-year	88.6%	84.2%	0.95^a^
	2-years	70.7%	70.3%	
Clinical benefit	1-year	87.1%	89.7%	<0.01^a^
	2-years	74.1%	82.5%	
ABI	1-year*	0.85 ± 0.18	0.86 ± 0.16	0.41
	2-years*	0.84 ± 0.21	0.85 ± 0.17	0.41

*TLR* target lesion revascularization and *ABI* ankle brachial index

*Statistically significant compared to pre-procedure, *p* < 0.01

^a^Log-rank *p* values comparing the two groups over time (i.e., from pre-procedure through 2 years)

^b^Three of the amputations in the CRF group and one amputation in the non-CRF group occurred prior to 2 months

### Patency and Clinical Outcomes

**Fig. 3:**
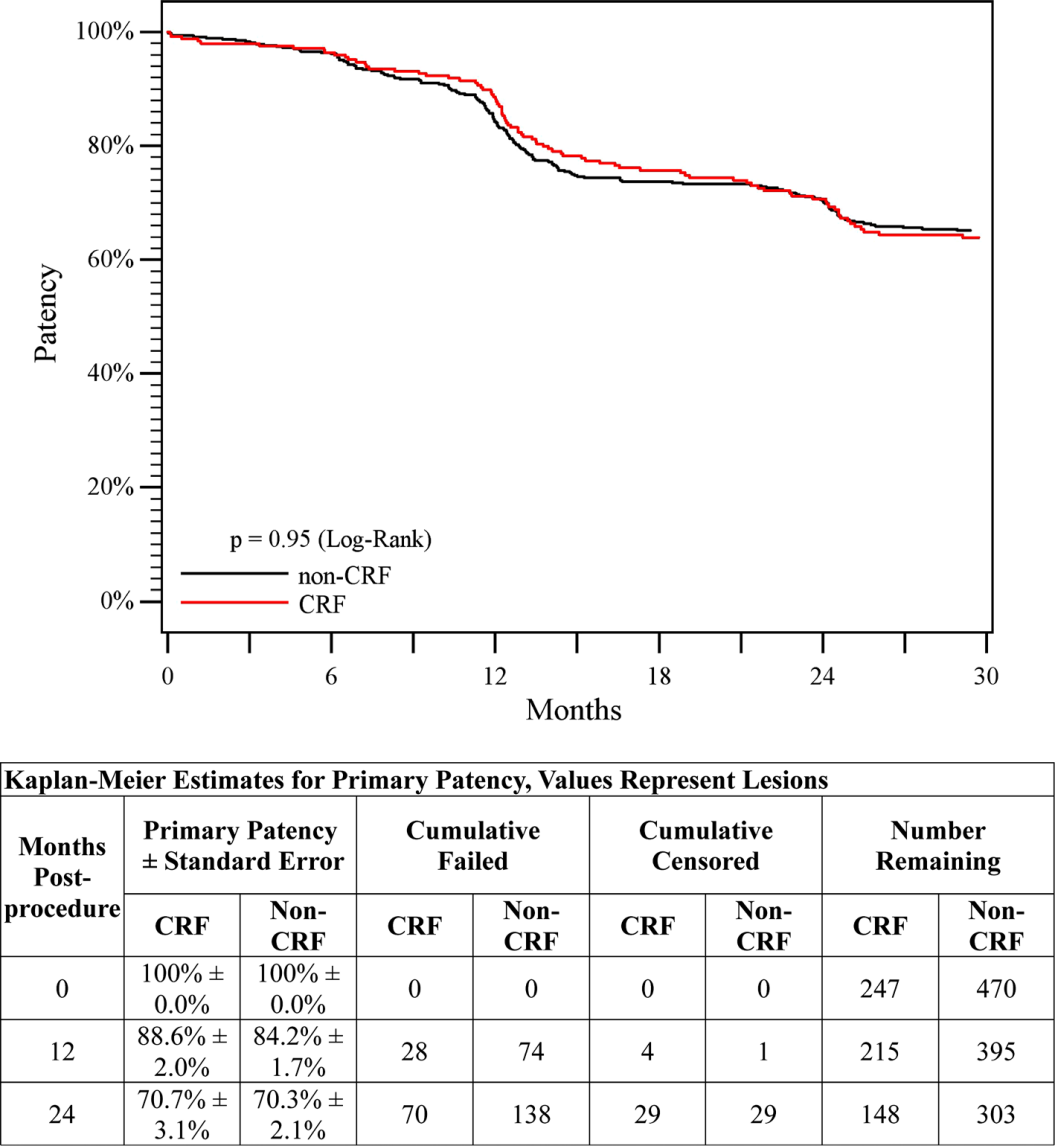
Primary patency. Kaplan–Meier curves of primary patency for patients in the non-CRF group (*black line*) versus patients in the CRF group (*red line*). Primary patency was 70.3% in the non-CRF group versus 70.7% in the CRF group through 2 years (*p* = 0.95, log-rank test)

**Fig. 4:**
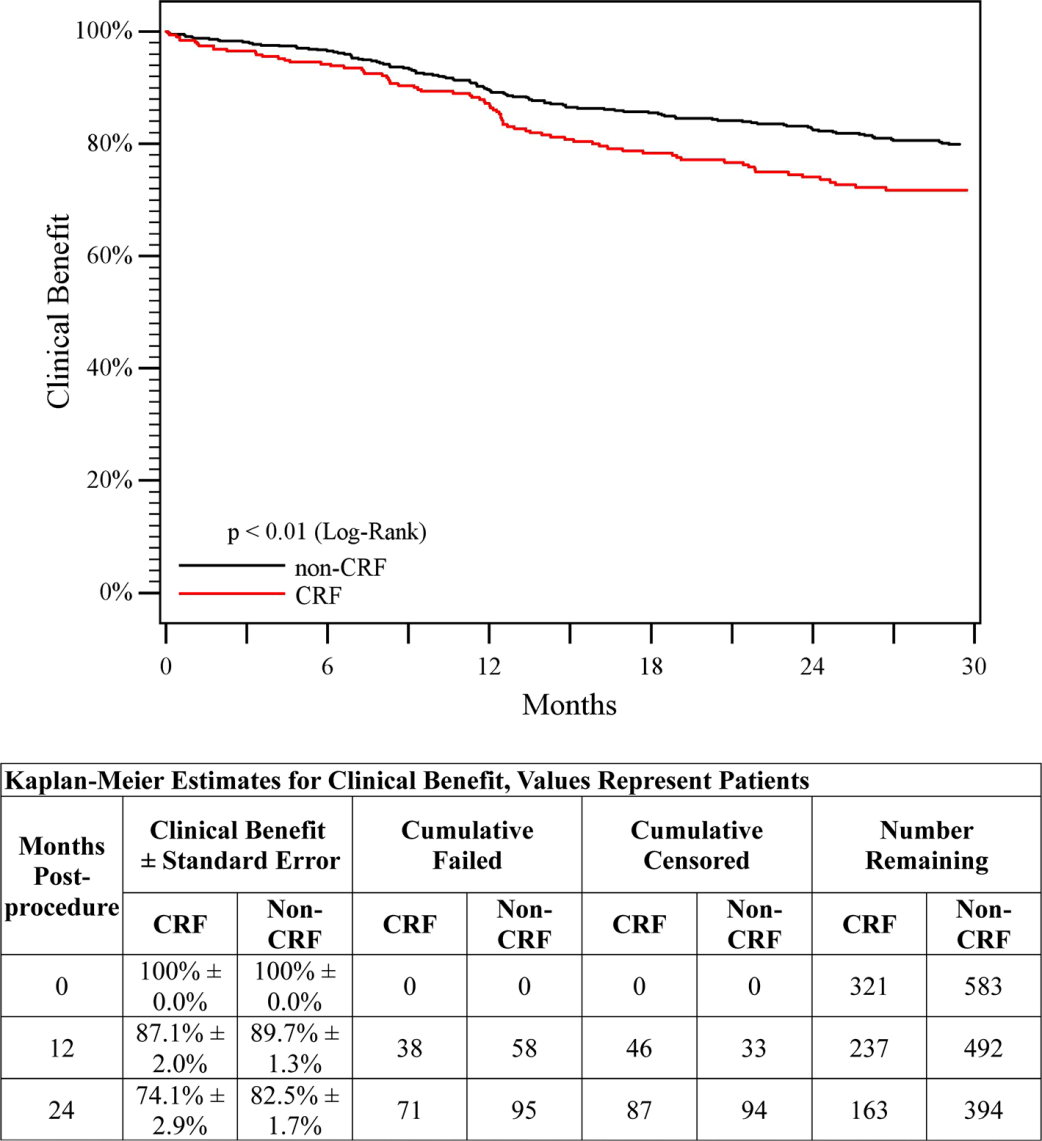
Post-treatment clinical benefit. Clinical benefit was defined as freedom from persistent or worsening symptoms of ischemia (i.e., claudication, rest pain, ulcer, or tissue loss) after the initial study treatment. Kaplan–Meier curves of clinical benefit for patients in the non-CRF group (*black line*) versus patients in the CRF group (*red line*). Clinical benefit was 82.5% in the non-CRF group versus 74.1% in the CRF group through 2 years (*p* < 0.01, log-rank test)

**Fig. 5:**
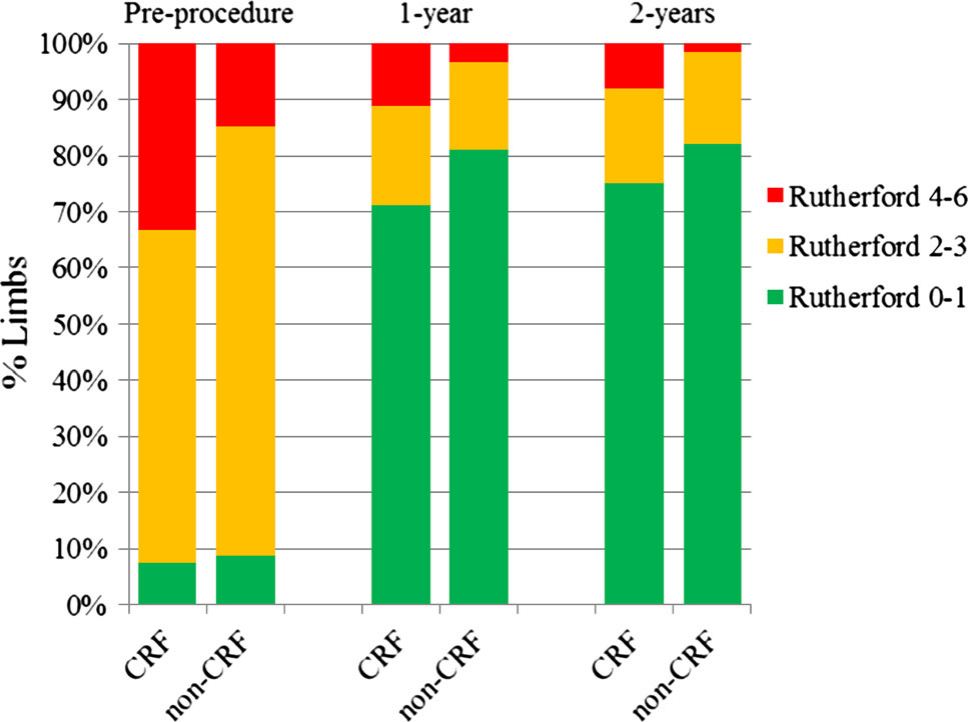
Rutherford classification. Rutherford classification significantly improved for both groups from pre-procedure (*p* < 0.001). The incidence of CLI was reduced through 1 and 2 years in both groups

Patency and clinical benefit results through 2 years are presented in Table 2. There were no significant differences in patency between the CRF and non-CRF groups through 2 years (70.7 versus 70.3%, *p* = 0.95 log-rank). The clinical benefit rates were similar in the CRF and non-CRF groups through 1 year; however, through 2 years, the rate in the CRF group was lower compared to the non-CRF group (74.1 versus 82.5%, *p* < 0.01 log-rank). Kaplan–Meier curves are provided in Figs. 3 and 4. As shown in Table 2, ABI improved in both groups from pre-procedure through 1 and 2 years. In addition, overall Rutherford classification improved and the incidence of CLI was reduced through 1 and 2 years in both groups (Fig. 5).

## Discussion

The present study demonstrates the safety and effectiveness of the DES for treatment of FP lesions in patients with CRF. To the best of our knowledge, this is the first comparative study of the DES for FP lesions between CRF and non-CRF groups.

Traditionally, CRF is considered a high-risk factor for restenosis, with twice the incidence of restenosis in CRF patients undergoing hemodialysis [5], and decreased limb salvage after endovascular therapy, with twice the rate of amputation in patients with severe CRF compared to patients with mild and moderate CRF [4]. CRF is commonly associated with PAD lesions that are more severely calcified and diffuse than those observed in patients without CRF [4, 5]. Pathologically, increased chronic inflammation is typically noted in the peripheral arteries of patients with CRF compared to the vessels of non-CRF patients with PAD. Furthermore, CRF patients are frequently on dialysis, which results in platelet dysfunction and may activate plasma coagulation factors that can cause restenosis due to resultant mural thrombosis [13–15].

CRF has also been identified as a risk factor for mortality in patients with PAD [2]. In the current study, mortality rates were three times higher in the CRF group compared to the non-CRF group. This is consistent with previous studies where the mortality rates have been reported as 13% for patients with mild CRF and 41% for patients with severe CRF [4].

In the present study, diabetes, critical limb ischemia, calcification, and reduced runoff were more frequently seen in the CRF group, and total occlusions were more prevalent in the non-CRF group. Contrary to traditional expectations and despite the differences in demographics and lesion characteristics, there were no significant differences in stent patency, TLR, and thrombosis between the CRF and non-CRF groups. Some previous BMS studies also demonstrated that stent patency was not affected by the presence of CRF [16–19]. However, these studies included only a small number of patients with renal failure. The results with drug-coated balloons in CRF patients have not been previously reported. The 2-year freedom from TLR rate in the CRF group was 81.4%, compared to 86.0% in the Zilver PTX Randomized Clinical Trial [9]. The 2-year stent patency rate was 70.7% in the CRF group compared to 74.8% in the Zilver PTX Randomized Clinical Trial [8]. One explanation for these differences could be that the CRF patients had a greater frequency of CLI and longer lesion lengths—over twice as long—when compared to what was reported in patients enrolled in other studies with this DES.

Despite no significant difference in stent patency or TLR between the two groups, the clinical benefit in the CRF group decreased around 1 year relative to the non-CRF group, primarily due to claudication and rest pain in the CRF group. Also, the 2-year clinical benefit rate in the CRF group (74.1%) was lower than the published rate of 81.8% for Zilver PTX in the Randomized Clinical Trial [8]. The relatively lower clinical benefit in CRF patients may be due to the nature of their underlying disease with poor tibial runoff and/or severe calcification, which could progress to a worsening clinical condition and Rutherford classification. Additional factors that accompany CRF, but are not related to stent performance, including malnutrition, non-healing advanced tissue loss, and immunologic dysfunction [4, 20, 21], likely also contribute to reduced clinical benefit. Many previous BMS studies have also shown the deleterious effect of CRF on clinical outcomes [1, 3, 4, 17, 19, 22].

Nearly all amputations occurred in patients with pre-procedure Rutherford classification of five. Additionally, three amputations in CRF patients, all with pre-procedure Rutherford classification of five, occurred within the first 2 months following DES placement, even though the stents remained patent through this time. This likely reflects the complex nature of CRF and a more advanced stage of PAD at the time of treatment, which may have resulted in a pre-procedure expectation of planned distal amputation. Although the 2-year rate of amputation in the CRF group (2.5%) was statistically higher than that observed in the non-CRF group (0.3%), these rates were both lower than the 5–28% amputation rates reported in previous BMS studies [4, 5, 18, 23]. In some surgical bypass studies, approximately half of patients with severe CRF required amputation despite patent bypass grafts because of the complex nature of CRF and high frequency of CLI [24, 25].

Also of note, CLI was reduced after DES placement in CRF patients to approximately one-fourth the pre-procedure frequency in the present study. Thus, DES treatment of CRF patients with FP PAD appears to provide beneficial outcomes compared with those achievable with BMS and other standard endovascular interventions, even in patients with CLI.

Although the stent fracture rates were low in both the CRF and non-CRF groups, fractures were more frequent in the non-CRF group. The FP arteries are exposed to various forces such as compression, torsion, or elongation [26], and those forces are known to cause stent fractures [19]. Also, the overall length of the stented FP segment has been shown to be associated with an increased risk of stent fractures [27]. The reason for the lower incidence of stent fractures in the CRF group is unclear. Possible explanations may be related to the hard calcified arterial walls routinely present in CRF patients which may resist the various forces responsible for stent fracture and/or the fact that the CRF group was comprised of more CLI patients whose restricted level of physical activity and ambulation may limit untoward forces upon FP stents to a greater degree than in non-CLI patients. Further long-term evaluation may be needed to better understand this possible difference between CRF and non-CRF patients.

This study has several limitations. As described in the original study, it is difficult to distinguish between stent thrombosis and total occlusion caused by restenosis in FP PAD patients because there is no standardized classification for superficial femoral artery stent thrombosis. As such, we relied on site-reported determinations of stent thrombosis. Unfortunately, differences among sites and investigators may result in variable diagnosis.

Duplex ultrasonography for evaluating stent patency was performed only at sites where it was considered standard of care during follow-up surveillance. Consequently, approximately one-third of stented lesions were not evaluated for patency and are therefore not included in the calculation of patency. The present study collected information regarding the presence of renal failure (defined as eGFR <60 mL/min/1.73 m^2^ and/or dialysis), but did not collect further information to distinguish if patients were receiving dialysis or experiencing severe CRF (eGFR <30 mL/min/1.73 m^2^). It is known that patients with severe CRF or dialysis have a poorer prognosis including decreased limb salvage than patients with less severe renal failure [4]. More detailed categorization of CRF into subgroups using a standardized chronic kidney disease classification [28] may help better evaluate the full range of CRF patients and their response to various endovascular FP interventions.

## Conclusion

This is a first comparative study of Zilver PTX between CRF and non-CRF groups. These results indicate that the DES placed in FP lesions of CRF patients is safe and effective with similar patency and TLR rates compared to patients without CRF.
